# Critical Environmental Injustice: A Case Study Approach to Understanding Disproportionate Exposure to Toxic Emissions

**DOI:** 10.3390/toxics12040295

**Published:** 2024-04-17

**Authors:** Clare E. B. Cannon

**Affiliations:** Department of Human Ecology, University of California, Davis, CA 95616, USA; cebcannon@ucdavis.edu

**Keywords:** environmental justice, critical environmental justice, toxic emissions, rural community, environmental exposure, community-based participatory action research

## Abstract

Environmental justice research has focused on the distribution of environmental inequalities, such as proximity to landfills, across the U.S. and globally. Background: Public health research and environmental health research, specifically, have focused on toxic exposure—encompassing individuals or communities that are disproportionately exposed to contaminants that are harmful or potentially harmful to them. Yet, little research has applied critical environmental justice theory—characterized by the idea that marginalized communities need to be treated as indispensable rather than disposable—to the study of toxic exposure. To fill this gap, the current paper offers a case study approach applying critical environmental justice theory to the study of disproportionate and unequal exposure to toxic contaminants. Methods: This case study is of Kettleman City, a rural, unincorporated community in the heart of California’s Central Valley (USA). This community experiences the co-location of environmental hazards, including residing at the intersection of two major highways and hosting a class I hazardous-waste landfill, which is one of the few licensed to accept PCBs. PCBs are a contaminant that has been linked with several adverse health outcomes, including cancers and low birthweight. Residents may also experience poor air quality from proximity to the highways. Results: This case highlights the uneven distribution of pollution and environmental degradation that may be shouldered by the community, along with their experiences of adverse health and social impacts. This analysis reveals the importance of incorporating a critical environmental justice perspective to unpack experiences of not only disproportionate exposure but also disproportionate procedural and recognitional inequality. Conclusions: This research highlights the untapped potential of environmental justice to catalyze exposure science in challenging the unequal distribution of contaminants.

## 1. Introduction

Increasingly, researchers are paying attention to issues and concerns of toxic emissions disproportionately experienced by some communities more than others. Environmental justice scholarship has a long tradition of investigating such uneven distributions of environmental harms, their drivers, and the accompanying adverse social and health outcomes (e.g., [1,2,3,4,5,6]). While environmental justice research has focused on such distributions, more recently, scholars have begun to also investigate procedural and recognitional forms of environmental injustice and their intersections [7,8]. Procedural justice refers to who gets to participate in processes of decision making that affect communities, while distributive justice refers to the distribution of environmental harms and benefits, and recognitional justice refers to acknowledging historical and contemporary systems of oppression. More recently, environmental justice scholars have been developing a theoretical approach, critical environmental justice, that aims to address pitfalls within environmental justice research. For example, critical environmental justice articulates a framework that seeks to analyze intersectional differences across scales [9,10]. The current research fills a knowledge gap in how we understand procedural, distributional, and recognitional justice outcomes through the prism of critical environmental justice theory for rural communities that may be at risk of experiencing toxic emissions. 

To advance research into environmental justice and toxic emissions, particularly in rural, underserved communities, the current paper employs a case study approach to a rural community experiencing multiple sources of potential toxic emissions. Kettleman City, California (USA), is a farmworker community in the agriculturally productive Central Valley. It hosts a class I hazardous-waste landfill, one of two that are operational in the state of California. The town lies at the intersections of two major highways, I-5 and CA-41. This case study approach uses archival documents to outline the kinds of toxic emissions the community has been subjected to since the landfill opened over 50 years ago. These data are complemented by a small-scale pilot study of air quality—measuring metals and elemental and total carbon—conducted with and in the community. The objective of this paper is to apply a critical environmental justice theoretical framework, characterized by four pillars—intersectional differences, multi-scalar approaches, transforming institutions, and promoting indispensability [10]—to this case study, providing examples of distributive, procedural, and recognitional injustices related to toxic emissions. Doing so enables a more complete understanding of the drivers and consequences of the disproportionate distribution of toxic emissions and their cumulative impacts. 

### 1.1. Literature Review

#### 1.1.1. Environmental Justice and Rural Communities

Environmental justice research and activism have been crucial in identifying and attempting to address environmental inequalities, particularly those related to toxic sources, emissions, and exposure (e.g., [11,12]). Environmental justice research has shown that such environmental inequalities are linked to racial/ethnic and socioeconomic inequities in the distribution of various environmental hazards and risks in the U.S., particularly across urban areas, with numerous studies identifying how and where environmental hazards are distributed unevenly across communities and places (e.g., [1,2,3,4,5]). One strand of environmental justice research has investigated the disproportionate impact of environmental injustice on rural communities, including fracking [13], coal impoundments [14] and production [15], and hazardous waste facilities [16]. In turn, studies of ruralness have broadened the environmental justice framework (e.g., [17]). Several studies indicate that racial/ethnic minorities and low-income individuals experience disproportionate residential exposure to toxic emissions such as air pollution [18]; proximity to hazardous waste, construction and demolition, and industrial and municipal landfills [6]; and toxic releases from industrial facilities [19]. Finally, environmental justice research has begun to investigate cumulative impacts from multiple co-located environmental hazards, particularly in rural areas, together with social vulnerability indicators [20,21,22]. The limited studies into environmental justice in rural communities highlights the need for more work in such spaces. The current paper adds to this body of scholarship by applying a critical environmental justice theoretical framework to the case of a rural community at risk of toxic emissions. 

#### 1.1.2. Critical Environmental Justice

Building on prior environmental justice scholarship and activism (e.g., [1,2,3,4,5]), critical environmental justice seeks to deepen approaches to entrenched inequalities by identifying the processes and systems that drive them (e.g., [9,10]). In articulating a critical environmental justice theoretical framework, Pellow [9,10] argues there are four pillars—expanding categories of difference, advancing multi-scalar approaches, identifying institutional inequalities, and promoting indispensability—that can help us to assess and address entrenched environmental inequalities. For instance, Pellow argues that expanding categories of difference in environmental justice analyses enables a framework for identifying intersectional systems and processes of oppression and their effects. The second pillar includes multi-scalar frames for understanding and analyzing injustices across expanded categories of difference. Multi-scalar frames provide the ability to analyze both the ways in which micro (e.g., small groups) and macro (e.g., global) spatial and temporal (e.g., historical forces) scales play vital roles in illuminating the experiences of environmental injustice. The third pillar highlights how environmental justice movements and advocates may need to reform or transform the institutions (e.g., the state, agencies, and NGOs) that produce and maintain inequalities. The fourth and final pillar shows that specific social groups have been marginalized and subjected to environmental injustices because they are perceived to be and treated as expendable. Scholars have begun applying this framework to a wide range of environmental justice problems, particularly in urban spaces, such as the inequality experienced by urban coyotes [23], the relationship between urban forests and race [24], and the relationship between residential segregation and urban tree canopies [25]. Whereas few studies have used this theory in studying toxic spaces (e.g., [26]), one novel contribution of the current paper is the application of this theoretical framework to understanding the procedural, distributive, and recognitional injustices a rural community faces with respect to risks of toxic emission exposure.

#### 1.1.3. Procedural, Distributive, and Recognitional Typologies of Environmental Justice

Some environmental justice research has used procedural, distributive, and recognitional justice to characterize different forms of injustice communities face [7]. Procedural justice refers to who gets to participate in processes of decision making that affect communities. EJ scholarship has worked to identify hidden and marginalized stakeholders and highlighted their inability to participate across a multitude of environmental issues [27], including access to clean drinking water (e.g., [28]) and local environmental decision making [29]. Communities’ ability to participate in meaningful solutions is essential for preventing their disproportionate exposure to toxics in the environment and to redressing such exposure after it has occurred. Distributive justice refers to the distribution of environmental harms and benefits [7]. Much EJ scholarship has focused on this kind of injustice, including the disproportionate distribution of environmental hazards such as landfills [30], hazardous industrial facilities [31], and poor air quality [12]. Such uneven distribution of hazards results in disproportionate exposure to toxics and its accompanying health impacts which tend to be experienced by marginalized communities (e.g., [12]). More recently, environmental justice scholars have called for recognitional justice, which asks whether identity and history have been acknowledged and included in environmental decision making [32]. Recognitional justice goes beyond the inclusion of communities in decision making that impacts their lives to address the larger systems and forces—such as the patriarchy, white supremacy, and classism—that have put communities in the position of experiencing procedural and distributive injustices in the first place. One novel contribution of this paper is applying the critical environmental justice framework to these environmental typologies of procedural, distributive, and recognitional justice.

## 2. Materials and Methods

### 2.1. Case Site: Kettleman City

**Figure 1:**
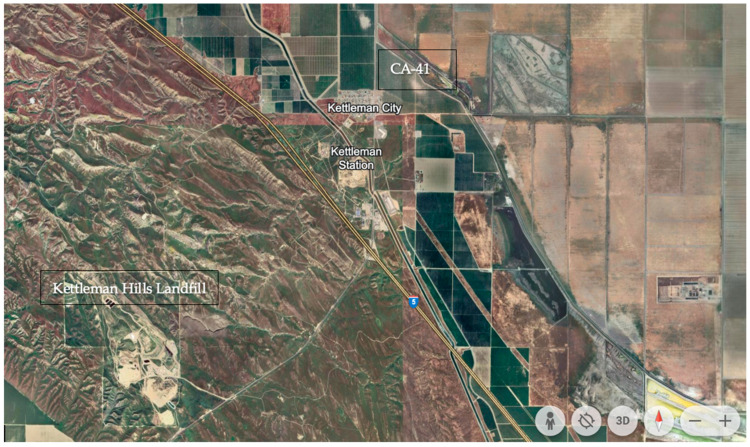
Satellite image of Kettleman City. Creative Commons: Google Earth.

Kettleman City, California, USA is a rural, unincorporated township located in the agriculturally productive Central Valley and hosts one of two operating class I hazardous-waste landfills in the state [33,34]. Class 1 hazardous waste facilities are permitted to accept solid, semi-solid, and liquid hazardous waste—waste defined as being harmful to human health or the environment—for final disposal [35]. Nestled in Kings County, Kettleman City was originally populated by oilfield workers following the discovery of oil in the area in the late 1920s [34]. Today, the town is located at the junction of two major highways, I-5 and CA-41, with the surrounding land used primarily for industrial agriculture, including pistachio, almond, and stone fruit production [36]. Kettleman City is a community of approximately 1200 residents, who mostly identify as Hispanic or Latinx (97.1% of residents identified as such). In 2019, 52% of the community identified as foreign-born, and 3 out of 4 foreign-born residents had yet to obtain U.S. citizenship [37]. Of the residents in the community, 28% reported incomes of less than 125% of the U.S. poverty level [37]. Figure 1 shows a satellite image of the area.

Given the study site’s location, there are multiple potential sources for toxic releases (i.e., landfill, highways). It was necessary to consider the cumulative environmental impacts of the multiple potential sources of emissions. To capture these impacts, a community-engaged small scale air quality monitoring project was conducted to identify whether there were toxic emissions that residents may be exposed to.

### 2.2. Case Study and Community-Engaged Research Approach

Research for this article was conducted between 2017 and 2023. Following a case study with a qualitative research design and analytic strategy (see [38,39,40]), a content analysis of historical and current documents taken from the U.S. EPA, California EPA, and news reports was conducted. The data were analyzed using an explanation-building technique, in which the research question was open-ended and the data analyzed to explain the research question [41]. Additionally, a pilot community-based participatory action research (CBPAR) project that partners with communities to address their concerns about toxics was conducted. CBPAR is a kind of research in which researchers partner with community organizations in order to conduct rigorous and relevant research with an extended reach for science, the community, and decisionmakers [42,43]. In this study, researchers partnered with El Pueblo para el Aire y Aqua Limpia de Kettleman City (El Pueblo), an environmental justice organization in Kettleman City, and Greenaction for Health and Environmental Justice (Greenaction), throughout the project—from identifying research questions to research design and data collection to the analysis and reporting of findings. For example, lifelong residents of the town described smelling pesticides sprayed on neighboring orchards, and had previously counted over 400 trucks per day on the CA-41 heading to the landfill. Based on these experiences and observations, and together with community partners, we designed the preliminary air quality sampling and analysis study described below.

### 2.3. Preliminary Study of Air Quality: Materials and Methods

Repeated violations at the landfill, its presence, and experiences in the neighboring community led to a landmark civil rights settlement in 2016 between both El Pueblo and Greenaction. There were no funds associated with this settlement, but the state committed to reviewing whether its decision for a permit renewal complied with civil rights laws, including greater public participation in the renewal process, ensuring better language access, and working with the community to further study the concerns raised in the lawsuit [44]. The current pilot study was an initial attempt to collect a small amount of data to begin identifying potential toxic air emissions impacting the community.

For this study, scientists worked with El Pueblo and Greenaction, who won the civil rights settlement discussed above. These community organizations were partners throughout the entire research project. Given community partners’ interest in the traffic they observed—including trucks to and from the landfill, they identified a need to measure black carbon, a carcinogen in the state of California, as an indicator for such pollution. To measure diesel exhaust pollution, four PM2.5 Interagency Monitoring of Protected Visual Environments (IMPROVE) monitors measured particulate matter sampled over 12 days. The IMPROVE samplers measured continuously for 72 h each over a two-week period, and all four modules ran simultaneously. The samplers were set up in the middle of town, two blocks from CA-41, 2 miles as the crow flies from the landfill and 1 mile from the I-5. IMPROVE samplers are used across a network to monitor outdoor air quality across the U.S., governed by a steering committee led by the U.S. EPA and National Park Service. As such, IMPROVE monitors have rigorous quality assurance and control (QA/QC) measures. (Full QA/QC measures can be found at https://vista.cira.colostate.edu/Improve/quality-assurance/ (accessed on 31 January 2024). Additional information on IMPROVE samplers can be found at https://vista.cira.colostate.edu/Improve/wp-content/uploads/2023/10/IMPROVE_Data_User_Guide_24October2023.pdf (accessed on 31 January 2024).) The sampling strategy was preliminary and a necessary first step in beginning to investigate the air quality of the community by answering calls that local community partners and residents had been making for several years.

Samples collected with IMPROVE samplers to identify and measure indicators of air pollution were analyzed using several protocols; Teflon filters were analyzed using X-ray fluorescence (XRF) to determine the elemental composition of air samples to determine the concentration and presence of heavy metals known to be dangerous to human health, such as mercury, arsenic, and lead; Teflon filters were analyzed using a hybrid integrating plate/sphere system (HIPS), an optical absorption measurement for black carbon, an indicator of diesel pollution and a designated carcinogen in the state of California; quartz filters were analyzed to provide an indication of organic and elemental carbon content (TOR). Elemental carbon is a measure of black carbon, an indicator of diesel pollution. Analyses were conducted by the UC Davis Air Quality Research Center.

The major goals of this preliminary approach to air quality monitoring were to answer calls from the community to begin sampling their air quality; provide a proof of concept for the sampling strategy; and generate pilot data to provide leverage for additional grants to develop a more robust, longer-term air monitoring program with the community.

## 3. Results

### 3.1. Case Study Results: Kettleman Hills Landfill

**Figure 2:**
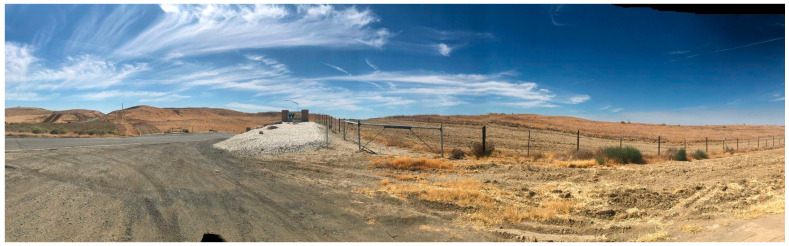
Kettleman Hills Landfill Entrance from CA-41 in 2019. Photo credit: author.

Located 3.5 miles southwest of Kettleman City is the Kettleman Hills Facility, a hazardous-waste landfill owned and operated by Chemical Waste Management, Inc. [45]. In 1975, the Kettleman Hills Facility was built and permitted for oilfield waste disposal, and, in 1977, it expanded to become a hazardous waste disposal landfill [41]. While the public notice given at the time the landfill was built met legal requirements, the notice did not meaningfully inform Kettleman City residents of the site’s intended use [11]. Instead, residents learned of the landfill five years later, when it received media attention for violating environmental laws and was required to pay millions in fines [11]. This lack of meaningful community engagement represents one form of procedural inequality residents faced. Figure 2 is an image of the landfill entrance.

Currently, the Kettleman Hills Facility is operating under a Hazardous Waste Facility Permit [45]. Within the 1600-acre facility, 695.5 acres of land are permitted for hazardous waste, including the disposal of PCBs [46]. PCBs have been linked to adverse health outcomes including birth defects and cancers [47,48]. Though the current permit, which was put into effect in 2003, expired in 2013, the landfill continues to operate with its renewal application materials under review at the California Department of Toxic Substances Control [45].

Landfills continue to be a major environmental hazard linked to water contamination, greenhouse gas emissions (e.g., methane), and the accumulation of toxics in human and natural ecosystems [49]. Hazardous-waste landfills are a form of environmental inequality potentiating adverse health outcomes disproportionately experienced by poor communities and communities of color (e.g., [1,2,3,4]) and by rural communities [5].

Over the years, multiple violations of both the Resource Conservation and Recovery Act (RCRA) and the Toxic Substances Control Act (TSCA) have occurred at the Kettleman Hills facility [46]. For example, in March 1988, one of the landfill units in the facility experienced a slope failure, displacing waste within the unit and resulting in tearing of the site lining [50]. In 2005, the facility violated RCRA standards through shortcomings in their practices of sampling and testing hazardous waste [46]. In 2010, an inspection found that the facility had failed to meet the standards for laboratory quality control, as well as failing to fully determine whether hazardous waste leachate met the standards prior to disposal in the land [46]. In 2013, the facility was penalized after failing to report 72 spills of hazardous waste between 2008 and 2012, with the largest spill measuring between 5 and 8 gallons of waste [46]. The facility has also been subject to TSCA violations, including in 2004, when it was revealed by the facility that lysimeters—devices used to measure soil and water balance—at one of the PCB units had not been monitored as required between 1996 and 2003 [46]. That same year, an investigation by the U.S. EPA found that lab instruments used to analyze PCBs were not correctly calibrated, which the facility remedied [46]. In another example, Kettleman Hills facility was penalized in 2010 after an inspection discovered incomplete manifests and container labels, the use of a building contaminated with PCBs, and improper PCB disposal as a result of leaks and spills [46]. Again, in 2012, the facility disclosed that leachate from its PCB landfill had not been tested before being disposed of [46].

Over the years, residents of Kettleman City have also experienced a variety of health issues. A study conducted by the California Department of Public Health (CDPH) in 2010 identified 11 babies with structural birth defects, born between 2007 and March of 2010, who were born to mothers who had lived in Kettleman City either during their pregnancy or at the time of birth [34]. Three of these infants passed away within a year of being born [34]. While the number of birth defects observed during this time was greater than expected based on previous years, the investigation was unable to identify a specific cause of these anomalies [34]. Other adverse health outcomes experienced in the area, according to county-level health data outlined in the 2020 Environmental Justice Analysis, which cites information from the California Environmental Health Tracking Program, include Kings County residents, who make increased emergency room visits related to asthma compared to the rest of the state [51].

The studies conducted by the state in 2010 do show some recognition on the part of the state for the repeated violations of the landfill and of the harm experienced by residents, regardless of whether or not that harm was caused by the landfill. At the same time, these repeated violations without significant enough consequences to prevent further violations show recognition inequality.

### 3.2. Preliminary Study of Air Quality: Results

Table 1 presents the measurements from the IMPROVE samplers in Kettleman City for this preliminary study and compares them to the closest established IMPROVE monitor in Fresno, California, also located in the Central Valley. This table summarizes TOR analyses that show that Kettleman City has higher levels of elemental and total organic carbon—one measure of diesel exhaust pollution—compared to Fresno.

XRF analyses were used to identify heavy metals in the air. No concentrations of heavy metals meaningfully exceeded similar values from the IMPROVE Fresno samples collected in August 2019. Table 2 presents the XRF analyses for the Kettleman City samples and the IMPROVE Network’s Fresno monitor.

**Figure 3:**
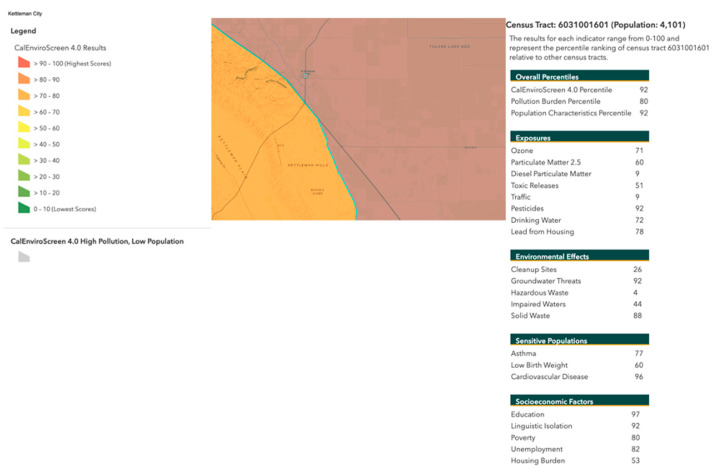
Screenshot of environmental impact percentile with cumulative impacts for Kettleman City, California, from CalEnviroScreen 4.0. The legend on the right shows the percentile for cumulative environmental and social impacts for the census tract of Kettleman City compared to all other California census tracts. The pie charts show the race/ethnicity and age profiles respectively.

Additionally, CalEnviroScreen 4.0—a data-screening tool used to aid in identifying California communities disproportionately burdened by multiple sources of pollution [51]—shows that Kettleman City is in the highest percentile for pollution compared to other California census tracts (see Figure 3). The tool includes exposure data (i.e., PM2.5, Ozone, diesel particulate matter, toxic releases, traffic, pesticides, drinking water, lead from houses), sensitive factors (i.e., asthma, low birthweight, cardiovascular disease), socioeconomic factors (i.e., education, linguistic isolation, poverty, unemployment, housing burden), and race/ethnicity.

### 3.3. Limitations

For air quality sampling, this pilot has several limitations. First, four samplers may not have been enough to identify the magnitude of the problem. Second, measuring for 72 h over two weeks may not have been frequent enough to capture the granularity of the emissions and to tie them to sources. Diesel particulates were not measured, and elemental and organic carbon are only indicators of diesel pollution. Lastly, elemental and organic carbon are just one measure of air pollution and do not address the multitude of hazardous air pollutants that may be in the air. Future research should seek to perform more robust air quality sampling to both identify substances that may be toxic in the air and to tie them to source emissions. For the particulate analyses, the sample was integrated over a long period of time (2 weeks), so short periods of high concentration when the wind may push clean air downstream may have diluted the pollution from sources of interest, making it difficult to measure the effects of these sources. For XRF analyses, a number of elements were targeted, but they were not exhaustive. There might be elements with accompanying adverse health outcomes that were not analyzed.

The results drawn from this research are constrained by the particularities of its context, though our findings may provide insights into experiences of marginalization and discrimination by those who experience toxic emissions firsthand. Additional research is needed to further test the insights garnered here to verify them and to identify whether they occur in other contexts. There are several limitations to our case study methodology. First, the evidence presented here may not be statistically generalizable but may be analytically generalizable. Analytic generalizations extend from establishing logic from this case that may apply to other similarly situated cases, such as other rural communities in California and beyond. Second, case studies are a form of data reduction; there are too many details to reproduce in their entirety. More research is needed to confirm the insights drawn here. The lessons taken from this research could assist in other community-engaged environmental justice research into toxic emissions across the U.S. and globally. Doing so could help fulfill calls by others in growing and expanding environmental justice research in the U.S. and internationally [52,53].

## 4. Discussion

The comparison between air quality samples—specifically carbon and heavy metals—taken from Kettleman City and the ongoing stationary monitor at Fresno is imperfect. Yet, it can provide a baseline of toxic emissions in the area relative to other places in the region. The limitations to this approach are discussed above along with calls for additional future research to test and extend the results found here. This analysis did reveal that Kettleman City has consistently higher total elemental carbon and organic carbon compared to Fresno (see Table 1), suggesting that there is a great deal of diesel pollution that is experienced. This is one example of distributional inequality—the greater levels of pollution experienced in Kettleman City. Diesel pollution has been associated with a number of adverse health outcomes, including cardiopulmonary death, hospitalizations for cardiovascular and pulmonary complaints, and emergency room visits for asthma exacerbations [54]. In the U.S., regulators only provide thresholds for diesel pollution in mines, and there is no permissible exposure limit for diesel exhaust pollution in the country [55]. Our preliminary sampling strategy was not able to identify the sources of the pollution. However, given the rurality of the community—its isolation from other sources—along with the traffic from the two major highways and the truck counts conducted by community partners, it seems that these may be the most likely sources of emissions. Moreover, the California Department of Transportation traffic data for Kettleman City in 2019 (the year’s samples were collected) at the CA-41 junction shows that there was an average annual daily traffic value of 40,000 [56]. Of this traffic, 10,604 vehicles were trucks, making up 26.51 percent of the daily traffic for that year on average [56].

Since landfills are the third largest producer of human-caused methane gas emissions in the U.S. [57] and have been linked to potential adverse health impacts, including cancers and asthma [58], it is important for additional research to both investigate other forms of air quality and attempt to locate sources of emissions. Moreover, landfills, as major sources of methane gas, are often overlooked contributors to climate change [59]. Thus, more research is needed both to understand the air quality of communities like Kettleman City that host such hazards, as well as their contributions to climate change.

This initial case study suggests that there may be disproportionate exposure to emissions from the landfill given its repeated failures to follow environmental laws. There also may be distributional inequality related to poor air quality—particularly diesel exhaust pollution—experienced by the community. This study was unable to definitively link poor air quality to source emissions, though the most probable source, given the high levels of elemental carbon, is the traffic on the intersecting highways, including traffic to and from a nearby distribution center and the landfill. More research is needed to identify the source of emissions. In this paper, measuring distribution of environmental harms in the community (i.e., landfill violations, poor air quality) was begun, which points towards distributive injustice—where environmental harms tend to be located or co-located together [7]. Meanwhile, a critical environmental justice approach helps researchers and communities to better understand why a community is not only disproportionately exposed to toxics (distributive), but also how procedural and recognitional injustice reinforce such unequal distributions.

In this section, the four pillars of Pellow’s critical environmental justice theory [10]—expanding categories of difference, multi-scalar frames, institutional transformation, and indispensability—are applied to the case of Kettleman City presented above to unpack the typologies of injustice and offer recommendations for advancing procedural, distributive, and recognitional justice.

Expanding the categories of difference provides a wider view of the intersectional processes that create and maintain oppression. For example, the intersection of ruralness, the unincorporated status of the town, and the majority Latinx population each contribute to the procedural injustice they face, which has implications for the uneven distribution of toxics they experience. As an unincorporated township, the community does not have a strong voice in local or county governance structures. Such a lack of inclusion and meaningful representation means that decisions happen without the community being able to voice their concerns, needs, or opinions [43]. Compounding this lack of political representation is the intersection with ruralness. Being in a rural area means there are additional barriers, such as transportation, to participating in governance processes related to environmental injustices [60,61]. As a majority Hispanic/Latinx population, residents also report feeling discriminated against because of their ethnicity [43]. The lack of procedural access contributes to the distribution of environmental harms in the community. For example, while the public notice that was given at the time the landfill was built met legal requirements, the notice did not meaningfully inform Kettleman City residents of the site’s intended use [11].

Multi-scalar approaches similarly widen available frames for identifying, analyzing, and addressing oppressions that are not constrained by time and space. For example, environmental justice research has shown that environmental harms tend to be co-located, such as the location of landfills, distribution centers, industrial hazards, etc. [1,4,5,20]. These co-located hazards can produce disproportionate distribution of toxic emissions that have cumulative impacts. Cumulative impacts are not just the individual impacts from these hazards, but also the additive or cascading impacts from the intersections of the hazards [20]. For example, a multi-scalar approach allows for this kind of pollution context as the sources of emission and social vulnerability are captured at different scales and data are collected and regulated by different agencies at different levels (i.e., state, federal). The impacts of such sources of toxic emissions are cumulative—across time and space—so must also be investigated and addressed multi-scalarly.

Critical environmental justice points towards the need for institutional transformation to advance environmental and social justice. Such transformation has implications for advancing procedural, distributive, and recognitional justice. In one example of institutional transformation, the state agency responsible for the landfill renewal, the DTSC, was recently overhauled as a result of the passage of Senate Bill (SB) 158 in 2021, with the implementation of a major environmental justice initiative, the Board of Environmental Safety (BES). This overhaul is a form of recognitional justice as it seeks to infuse environmental justice throughout the agency as a reaction to the historical environmental injustice created by the agency’s decision making. The BES is a five-member board with an ombuds program and Environmental Justice Advisory Council to advise the board on its decision making (SB 158). This advisory council is one mechanism for advancing procedural justice by having community members work within agency structures to work towards reducing environmental injustice. The BES itself hears and decides on appeals on permitting decisions, such as the one pertaining to the Kettleman Hills facility; sets rates for certain fees; and reviews the agency’s programs in relation to other agencies (such as the California Air Resources Board) for redundancies (SB 158). With the recent creation of this board, it remains to be seen if the distributive outcomes for Kettleman City residents will improve.

Finally, the fourth pillar of critical environmental justice argues that historically marginalized and excluded peoples have been treated as dispensable, as have the lands they live, work, and play on. To rectify environmental injustice, the critical environmental justice approach argues these same communities must be understood and treated as indispensable [9,10]. For example, the civil rights settlement that the Kettleman City activists won calls for additional mechanisms that increase procedural justice, including greater public participation in the landfill renewal process and better language access [44]. Advances in procedural justice can have knock-on effects for advancing recognitional and distributive justice (and vice versa). The civil rights settlement also points towards recognitional justice through the recognition of the larger social structures that have contributed to the disproportionate distribution of toxic emissions and related potential exposure in the community.

Bringing these insights together, critical environmental justice with its four pillars—intersectional differences, multi-scalar frames, transforming institutions, and promoting indispensability—is key to both identifying examples of procedural, distributive, and recognitional injustice, particularly around toxic emissions, and providing a way forward for advancing justice in communities like Kettleman City. We see examples of this in a community response of resilience and uplift, with residents fighting for their rights to an environment that will not harm them [43]. The civil rights suit is one example of such a community response. The continued organizing, activism, and advocacy to both stop additional environmental hazards with their toxic emissions, like the re-permitting of the landfill, and calls on the local and state government to invest funds to remediate the harm that has been inflicted for nearly 50 years is another example of the community fighting back [43]. The struggle for justice from toxic emissions continues.

## 5. Conclusions

In this paper, instances of distributive, procedural, and recognitional injustice have been explored in the case of Kettleman City, California (USA), a rural, unincorporated township with a predominantly Hispanic/Latinx community in the heart of California’s Central Valley. This study applied a critical environmental justice framework to identify examples of procedural, distributive, and recognitional injustice to a case study with preliminary study results from small-scale air quality monitoring. Critical environmental justice theory posits four pillars necessary to advance justice—intersectional differences, multi-scalar frames, transforming institutions, and promoting indispensability [9,10]. This framework enables us to examine not just the disproportionate distribution of toxic emissions, but also how such distributive injustice is enabled and maintained through procedural and recognitional injustice. This theory also provides a framework for identifying the ways communities like Kettleman City are fighting back.

Finally, this research opens additional avenues of exploration. For example, more research is needed to empirically assess both the mechanisms and outcomes of procedural and recognitional injustices, particularly for rural communities on the frontlines of environmental injustice, including those who experience disproportionate exposure to toxics. Future research could consider using critical environmental justice in its approach to studying toxic emissions, including the measurement and analysis of not just air pollution but also water and soil pollution. This study has begun to link together key concerns across different kinds of injustice (i.e., procedural, distributive, and recognitional) and critical environmental justice to better explain the context and drivers of the distribution of toxic emissions. Doing so will hopefully inspire others to consider environmental justice in research into toxics.

## Figures and Tables

**Table 1:** Measurement of total elemental carbon and total organic carbon from the IMPROVE sampler stationed in Fresno from August 2019 and results from TOR analyses used to measure elemental and organic carbon in samples taken from IMPROVE samplers used in Kettleman City in August 2019.

Site Code	Date	Total Elemental Carbon	Total Organic Carbon	Site Code	Date	Total Elemental Carbon	Total Organic Carbon Total
FRES1	08/10/2019	0.07	2.13	KC Pos 1	8/8/19–8/10/19	4.63	47.01
FRES1	08/13/2019	0.48	2.49	KC Pos 2	8/11/19–8/13/19	3.72	41.79
FRES1	08/16/2019	0.403	2.31	KC Pos 3	8/14/19–8/16/19	6.59	64.69
FRES1	08/19/2019	0.26	1.4	KC Pos 4	8/17/19–8/19/19	3.15	35.67

Note: Fresno measurements taken from Federal Land Manager Environmental Database (2019) (Federal Land Manager Environmental Database, Colorado State University; accessed via https://views.cira.colostate.edu/fed/Pub/DatasetDetail.aspx?dssl=1&dsidse=10001 (accessed on 31 January 2024). IMPROVE is a collaborative association of state, tribal, and federal agencies and international partners. The U.S. Environmental Protection Agency is the primary funding source, with contracting and research support from the National Park Service. The Air Quality Group at the University of California, Davis, is the central analytical laboratory, with ion analysis provided by the Research Triangle Institute, and carbon analysis provided by the Desert Research Institute.

**Table 2:** Metals identified in XRF analyses for Kettleman City samples compared to IMPROVE network’s Fresno samples in mass concentration per air volume measured (µg/m^3^).

Site Code	Date	Ni	Mg	Al	Si	As	Zn	Cr	K	Cu	Ti	Mn	Fe	Pb
KC Pos 1	8/8/19–8/10/19	0.000	0.060	0.198	0.597	0.000	0.003	0.001	0.083	0.001	0.015	0.005	0.199	0.000
FRES1	08/10/2019	0.000	0.006	0.071	0.167	0.000	0.002	0.000	0.078	0.002	0.005	0.002	0.062	0.000
KC Pos 2	8/11/19–8/13/19	0.001	0.084	0.286	0.856	0.000	0.004	0.001	0.117	0.002	0.021	0.007	0.302	0.001
FRES1	08/13/2019	0.000	0.023	0.17	0.428	0.000	0.006	0.000	0.132	0.011	0.014	0.003	0.17	0.000
KC Pos 3	8/14/19–8/16/19	0.000	0.084	0.227	0.693	0.000	0.002	0.001	0.100	0.001	0.017	0.005	0.226	0.000
FRES1	08/16/2019	0.000	0.063	0.191	0.52	0.000	0.005	0.001	0.097	0.006	0.016	0.005	0.208	0.001
KC Pos 4	8/17/19–8/19/19	0.000	0.061	0.164	0.491	0.000	0.003	0.001	0.094	0.001	0.012	0.004	0.162	0.000
FRES1	08/19/2019	0.000	0.041	0.172	0.423	0.000	0.003	0.000	0.074	0.002	0.012	0.003	0.155	0.000

## Data Availability

Data are contained within the article.
